# Characterization and Comparative Analysis of the Staphylococcus aureus Genomic Island *v*Saβ: an *In Silico* Approach

**DOI:** 10.1128/JB.00777-18

**Published:** 2019-10-21

**Authors:** Anita J. Kläui, Renate Boss, Hans U. Graber

**Affiliations:** a Food Microbial Systems, Agroscope, Bern, Switzerland; b Risk Assessment Division, Federal Food Safety and Veterinary Office, Bern, Switzerland; University of Illinois at Chicago

**Keywords:** *Staphylococcus aureus*, genomic islands, *in silico* characterization, comparative analysis

## Abstract

Staphylococcus aureus is a widespread opportunistic pathogen to humans and animals. Of its genome, 20 to 25% varies between strains and consists of phages, pathogenicity islands, transposons, and genomic islands. S. aureus harbors up to three genomic islands, *v*Saα, *v*Saβ, and *v*Saγ. The *v*Saβ region of S. aureus can encode a number of virulence-associated factors, such as serine proteases, leukocidins, enterotoxins, bacteriocins, or a hyaluronate lyase. In this study, the *v*Saβ regions of 103 clinically relevant S. aureus strains were characterized *in silico* and compared to the three predefined *v*Saβ types. We here suggest a superordinate system of 15 different *v*Saβ types, of which 12 were newly defined. Each *v*Saβ type has a distinct structure with a distinct set of genes, which are both highly conserved. Between the different types, gene content and composition vary substantially. Based on our data, a strain’s *v*Saβ type is strongly coupled with its clonal complex, suggesting that *v*Saβ was acquired in an ancestral S. aureus strain, arguably by phage mediation, before differentiation into clonal complexes. In addition, we addressed the issue of ambiguous nomenclature in the serine protease gene cluster and propose a novel, phylogeny-based nomenclature of the cluster contained in the *v*Saβ region.

**IMPORTANCE** With the rapid increase of available sequencing data on clinically relevant bacterial species such as S. aureus, the genomic basis of clinical phenotypes can be investigated in much more detail, allowing a much deeper understanding of the mechanisms involved in disease. We characterized in detail the S. aureus genomic island *v*Saβ and defined a superordinate system to categorize S. aureus strains based on their *v*Saβ type, providing information about the strains’ virulence-associated genes and clinical potential.

## Abstract (precis)

With the rapid increase of available sequencing data on clinically relevant bacterial species such as S. aureus, the genomic basis of clinical phenotypes can be investigated in much more detail, allowing a much deeper understanding of the mechanisms involved in disease. We characterized in detail the S. aureus genomic island *v*Saβ and defined a superordinate system to categorize S. aureus strains based on their *v*Saβ type, providing information about the strains’ virulence-associated genes and clinical potential.

## INTRODUCTION

Staphylococcus aureus is a commensal colonizer of the skin and mucous tissue of up to two-thirds of the human population (1, 2). It is an opportunistic pathogen in humans and animal hosts (3, 4), and its pathogenic potential ranges from being relatively harmless to causing potentially deadly infections (5). The S. aureus genome consists of the core genome and the accessory (or auxiliary) genome. The core genome, representing about 75%, is conserved in all S. aureus strains and consists largely of genes related to metabolism or other housekeeping functions. The accessory genome, about 20 to 25%, consists of mobile (or formerly mobile) genetic elements (MGE), such as bacteriophages, chromosomal cassettes, pathogenicity islands, transposons, and genomic islands (6). Its genes differ between strains (7–9) and are often related to virulence and antibiotic resistance. Therefore, they have important clinical implications, especially when considering their potential to spread horizontally (6, 7). The combination of these variable regions is the key to the high phenotypic variability in S. aureus.

S. aureus strains carry three different classes of genomic islands, *v*Saα, *v*Saβ, and the substantially smaller *v*Saγ. These genomic islands are extremely stable and have highly conserved genes. However, the gene compositions of each of the islands can vary substantially between some strains and are yet identical between others (8, 10).

In 2008, when Baba et al. (8) classified 12 sequenced S. aureus strains into 4 *v*Saα types and 3 *v*Saβ types, the comparison of the genomic islands was rather limited. To date, over 10,000 S. aureus whole genomes (including contigs and scaffolds) are available in the NCBI database. Such an enormous increase in sequencing data calls for a new, superordinate system to classify S. aureus strains based on genomic islands. The genomic island *v*Saβ is of particular interest, as it carries two genes belonging to the type I staphylococcal restriction-modification (RM) system (*hsdM* and *hsdS*) and harbors a number of virulence-associated genes, such as a hyaluronate lyase precursor gene (*hysA*), a lantibiotic gene cluster (bacteriocins of S. aureus [*bsa*]), two leukocidin genes (*lukD* and *lukE*), an enterotoxin gene cluster (EGC), and a cluster of serine protease genes (serine protease like [*spl*] genes).

First characterized in 2001, the *spl* cluster was described as an operon containing 6 genes (*splA*, *splB*, *splC*, *splD*, *splE*, and *splF*) with DNA sequence similarities ranging from 42% to 94% (11). For listing new *spl* genes, the existing alphabetical nomenclature is not ideal. Therefore, we establish an approach to unambiguously name the genes in the *spl* cluster, including new ones, based on their phylogenetic relationships.

To our knowledge, the findings of Baba et al. (8) have not been proceeded. Hence, we extended their *v*Saβ listing to a total number of 15 types by adding 12 new types obtained by analysis of the *v*Saβ islands of 103 clinical S. aureus strains. Thus, striking conservation of the virulence-associated genes was found within each type.

## RESULTS

Of the 103 analyzed strains, 37 strains showed known *v*Saβ types (I to III), and 66 strains showed novel *v*Saβ types (IV to XV) (see Table 1; also see File S2 in the supplemental material).

**TABLE 1:** Amino acid sequence identity of *v*Saβ-encoded proteins^*a*^

*v*Saβ type	Strain	Amino acid identity (%) for protein:																																	
		Sav1803	HysA	HsdS	HsdM	SplD2 (2)	SplD1	SplD2	SplD3	SplD4	SplC	SplB	SplA	Bla	BsaG	BsaE	BsaF	BsaP	BsaD	BsaC	BsaB	BasA2	BasA1	LukD	LukE	Seg	Sen	Seu	ϕent2	ϕent1	Sei	Sem	Seo	tRNA cluster	Sav1831
I	Mu50 (ref.)	+		+	+	+		+			+	+	+	+	*									+	+	+	+		+	+	+	+	+	+	+
	N315	100		99	100	100		100			100	100	100	100	100*									100	100	100	100		100	100	100	100	100	100	100
	502A	100		99	100	100		100			100	100	100	100	100*									100	100	100	100		100	100	100	100	100	100	100
	HOU1444-VR	100		99	100	99		100			99	100	100	100	100*									100	100	100	100		100	100	100	100	100	100	100
	JH1	100		99	100			97			100	100	100	100	100*									100	99	100	100		100	100	100	100	100	100	100
	JH9	100		99	100			97			100	100	100	100	100*									100	99	100	100		100	100	100	100	100	100	100
	Lodi13K	100		87	84			83			100	100	100	100	99*									100	100	100	100		100	100	100	99	100	100	100
	Mu3	100		100	100	100		100			100	100	100	100	100*									100	100	100	100		100	100	100	100	100	100	100
	NZAK3	100		99	100	100		100			100	100	100	100	100*									100	100	100	100		100	100	100	100	100	100	100
	UCI28	100		99	100	100		22*			100	100	100	100	100*									100	100	100	100		100	100	100	100	100	100	100
	ST228	100		99	100	100		46*			99	100	99	100	100*									100	100	100	100		100	100	100	100	100	100	100
II	TW20 (ref.)	+		+	+	+	+	+			+	+	+	+	+	+	+	+	+	+	58*	+	+	+	+									+	+
	COL	100		100	100	99	100	100			100	100	98	100	100	100	100	100	100	99	99	100	70*	100	99									100	100
	DSM 20231^T^	100		99	99	100	100	100			99	100	99	100	100	100	100	100	100	100	R	100	100	100	13*									100	99
	G12B	100		100	94	99	100	100			99	100	100	100	100	77*	100	100	100	100	100	100	100	100	99									100	100
	G29N	100		100	99	99	100	100			99	100	100	100	100	100	100	100	100	100	100	100	97	100	99									100	100
	Lodi10B	100		100	51*	99	100	100			99	100	100	100	100	77*	100	99	100	100	100	100	100	100	99									100	100
	M2084B	100		100	5*	99	100	100			99	100	100	100	100	77*	100	100	100	100	100	100	100	100	99									100	100
	M2130B	100		100	8*	99	100	100			99	100	100	100	100	77*	100	100	100	100	100	100	100	100	99									100	100
	M2529B	100		100	8*	99	100	100			99	100	100	100	100	77*	100	100	100	100	100	100	100	100	99									100	100
	M5512B	100		100	99	99	100	100			99	100	100	100	100	77*	100	100	100	100	100	100	100	100	99									100	100
	M5171B	100		100	100	99	100	100			99	100	100	100	100	77*	100	100	100	100	100	100	100	100	13*									100	100
	M6020B	100		16*	19*	99	100	100			99	100	100	100	100	77*	100	100	100	100	100	100	99	100	99									100	100
	MSSA476	90		99	99			99			98	99	99	99	100	77*	100	100	100	100	100	100	100	100	99									99	100
	MW2	90		99	99			99			99	99	99	100	100	100	100	100	100	100	99	100	100	100	99									99	100
	NCTC8325	100		99	99	100	100	100			100	100	100	100	100	100	100	100	100	100	100	100	100	100	13*									100	99
	Newman	100		100	100	100	100	100			100	100	98	99	100	100	100	100	100	100	100	100	100	100	99									100	100
	USA300	100		100	100	100	100	100			100	100	100	100	100	100	100	100	100	100	100	100	100	100	99									99	100
III	MRSA252 (ref.)	+	+	+	+		+	*	*	+	+	*														+	+	+			+	+	+	+	+
	55/2053	100	99	100	73*		100	R	R	100	100	100*														100	100	100			100	99	100	100	100
	A017934/97	100	100	100	100		100	47*	100	100	100	100*														100	100	100			100	100	100	100	100
	CIG1605	100	100	100	100		100	47*	17*	100	100	100*														100	100	100			100	100	100	100	100
	ATCC 25923	99	24*	100	100		100	100	100	100	99	100*														100	100	100			100	100	100	100	100
	ST1632	100	100	100	100		100	47*	17*	100	100	100*														100	100	100			100	100	100	100	100
	ST2788	100	100	100	100		100	47*	17*	100	100	100														100	100	100			100	100	100	100	100
	HAR21	100	100	100	100		100	47*	17*	100	100	100*														100	100	100			100	100	99	100	100
	TCH60	100	99	100	40*		99	100	99	99	100	100*														100	100	100			100	100	100	100	100
IV	M3783C (ref.)	+	+	*	*			+		+	+	+	*	+	+	+	+	+	+	+	+	+	+	+	+	*	+	+			+	*	+	+	+
	RF122	100	100	100*	100*			100		100	100	100	99*	99	100	100	100	100	100	100	99	100	100	100	99	100*	100	100			99	100*	100	100	100
	M2839C	100	100					100		100	100	100	100*	99	100	100	100	99	100	100	100	100	100	100	100	100*	87	100			100	100*	100	100	100
	M1280C	100	100	100*				100		100	100	100	100*	99	99	100	100	100	100	100	99	100	100	100	100	100*	100	100			100	100*	100	99	100
	M1655C	100	100	100*				99		100	100	100	100*	99	100	100	100	100	100	100	100	100	100	100	100	100*	100	100			100	100*	100	99	100
	M2323C	100	100	100*				100		100	100	100	100*	99	100	100	100	100	100	100	99	100	100	100	100	100*	100	100			100	100*	100	100	100
	M2682A	100	100	100*	100*			100		100	100	100	100*	99	100	100	100	95	100	100	100	100	100	100	100	100*	100	100			100	100*	100	80	65
	Sa110	100	100	15*				100		100	100	100	99*	99	100	100	100	100	100	100	100	100	100	99	99	100*	100	100			99	100*	100		
V	S0385 (ref.)	+	+																															+	+
	GD705	100	100																															100	100
	GD1677	100	99																															100	100
	NZ15	100	100																															100	29
	RVIM	100	100																															100	100
	UB08	100	100																															100	100
	USA7	100	100																															100	100
	VET030	100	100																															100	100
	08BA	100	100																															100	100
VI	K2R (ref.)	+	+	+	+	+	+	+			+	+	+	+	*									+	+									+	+
	STDY-6124959	98	100	98	99	100	100	100			100	100	100	100	100*									100	100									100	100
	HST-066	98	100	98	99	100	100	100			100	100	100	100	100*									100	100									100	100
	M0443	98	100	98	99	100	100	100			100	100	100	100	100*									100	14*									100	14
	1608 S21	100	100	100	100	100	100	100			99	100	100	100	100*									100	100									100	100
	st1012	98	100	98	99	100	100	100			100	100	100	100	100*									100	100									100	100
	STDY-6124877	98	99	98	99	100	100	100			100	100	100	100	100*									100	100									100	100
	Newbould	99	100	99	99	100	100	100			61	100	100	100	100*									100	14*									100	100
	G07I	99	100	99	99	100					100	100	100	100	100*									100	14*									100	100
	ATCC 6538	99	99	99	99	100	100	100			100	100	100	100	100*									100	100									100	100
	Lodi4R	99	*	100	99	97					100	99	100	100	100*									100	14*									100	100
VII	M013 (ref.)	+	+	*																														+	+
	M3386D	100	100	100*																														100	100
	MS4	100	100	100*																														100	100
	SA957	100	100	99*																														100	100
	HZW450	100	100	99*																														100	100
VIII	JKD6159 (ref.)	+	+	+	+		+			+	+	+	*	+	*									+	+									+	+
IX	ED133 (ref.)	+		+	+			+			+	+	*	+	+	+	+	+	+	+	+	+	+	+	*									+	+
	G42G	100		100	80			100			100	100	99*	100	100	100	100	100	100	100	100	100	100	100	100*									100	100
	NCTC1803	100		100	100			100			100	100	99*	100	100	100	100	100	100	100	100	100	100	100	100*									100	100
	NCTC9555	100		100	100			100			100	100	99*	100	100	100	100	100	100	100	100	100	100	57*	100*									100	100
	O267	100		100	99			100			100	100	99*	100	100	100	100	100	100	100	100	100	100	100	100*									100	100
X	O11 (ref.)	+		+	*		+	+			+	+^*b*^	+	+	*									+	*									*	+
	O46	99		100	100*			93			100	100	100	100	99*									98	R									*	100
XI	G33O (ref.)	+	*	+	*			+	+	+	+	+	+	+	*									+	+	+	*	+			+	+	+	+	+
	G68P	100	100*	100	99*			100	100	100	100	100	100	99	100*									100	100	100	99*	100			100	100	100	100	99
XII	G11F (ref.)	+		+	*		+	+	+	+^*c*^	+	+	+	+	*									+	+	+	+	+			+	+	+	+	+
	Strain 9	100		99	100*		100	100	100	100	100	100	100	99	100*									100	99	98	100	100			100	100	99	100	100
	FKQO	100		100	100*		100	100	100	100	100	100	100	99	100*									100	100	100	100	100			100	100	99	100	100
	GUATP222	100		100	100*		100	100	100	100	100	100	100	98	100*									100	100	100	100	100			100	45*	99	100	100
	GUATP47	100		100	100*		100	100	100	100	100	100	100	98	100*									100	100	100	100	100			100	100	99	100	100
	Ex1 scf	100		100	100*		100	100	100	100	100	100	100	99	100*									100	100	100	100	100			100	100	99	100	100
XIII	G19F (ref.)			+	+									+												+	+	+			+	+	+	+	+
	st1332			100	100									100												100	100	100			99	100	100	100	100
	NCTC8765			100	100									100												99	100	100			99	100	100	100	100
	MSSA			100	99									100												99	100	100			99	100	100	100	100
	strainNA			100	99									100												99	100	100			99	100	100	100	100
	TSAR05			100	99									100												99	100	100			99	100	100	100	100
	21334			100	99									100												99	100	100			99	100	100	100	100
	K18			100	100									100												50*	100	100			99	100	100	100	100
	RKI4			100	100									100												100	100	100			100	100	100	100	100
XIV	G08M (ref.)	+	+	+	*		+	+		+	+	+	+	+	*									+	+									+	+
	08-02300	100	100	100	R		100	100		100	100	100	100	100	99*									100	100									100	100
	3688STDY6125053	100	100	100	100		100	100		100	100	100	100	100	99*									100	100									100	100
	3688STDY6125046	100	100	100	100		100	100		100	100	100	100	100	99*									100	100									100	100
	H1524	100	100	100	91*		100	100		100	100	100	100	100	99*									100	100									100	100
	H1645	100	100	100	35*		100	100		100	100	100	100	100	99*									100	100									100	100
XV	Lodi11bM (ref.)	+		+	+	+	+	+	+	+	+	+^*b*^	+	+	*									+	+									+	+
	4185	100		99	99	100	100	100	99	84	100	100	100	99	98*									100	14*									100	100

a For each *v*Saβ type, a reference (ref.) strain was chosen. The *v*Saβ genes were translated into protein sequences using the standard code and aligned to the corresponding protein of the reference strain. Shading indicates identity of ≥95%, and asterisks indicate truncated or fragmented genes. Where a gene was absent or truncated in the reference strain, the protein sequence of another strain of that type was used as a reference, indicated with R below the corresponding protein. +, presence of the corresponding genes in the reference strains that served as a target for comparing the genes of the other strains of the group.

b *v*Saβ types X and XV have a second copy of SplB.

c *v*Saβ type XII has a second copy of SplD4.

*v*Saβ type I (*n* = 11) originated mostly from humans, with the exception of 1 strain originating from a bovine mammary infection. *v*Saβ type I is found commonly and included well-studied clinical strains, such as strains N315, 502A, Mu3, Mu50, and JH1. *v*Saβ type II (*n* = 17) comprised 8 human isolates with the well-known strain COL and strain DSM 20231^T^. The remaining isolates originated from bovine mastitis. Of the *v*Saβ type III strains (*n* = 9), 7 originated from humans (including the hospital-acquired MRSA252 and the quality control strain ATCC 25923), and 2 strains were of unknown origin. All *v*Saβ type IV strains (*n* = 8) were isolated from bovine mastitis. Of *v*Saβ type V (*n* = 9), one host was unknown, and the other 8 strains originated from humans. *v*Saβ type VI (*n* = 11) were all isolated from either humans or cattle.

Only a few strains harbored *v*Saβ types VII to XI. *v*Saβ type VII (*n* = 5) contained 4 human isolates and 1 bovine mastitis isolate, and for *v*Saβ type VIII, only a single isolate was found. *v*Saβ types IX (*n* = 5) and X (*n* = 2) were uniquely isolated from ovine mastitis. The two vSaβ type XI strains were bovine mastitis strains. From *v*Saβ type XII strains (*n* = 6), 2 originated from cattle and 4 from humans. Out of 9 vSaβ type XIII strains, 5 originated from human hosts and 2 from cattle, and for 2 strains, no information was available. One *v*Saβ type XIV (*n* = 6) strain was isolated from Swiss cattle and 5 from human hosts. The two strains with type XV were both bovine mastitis isolates.

### Structure of *v*Saβ and highly conserved regions.

**FIG 1:**
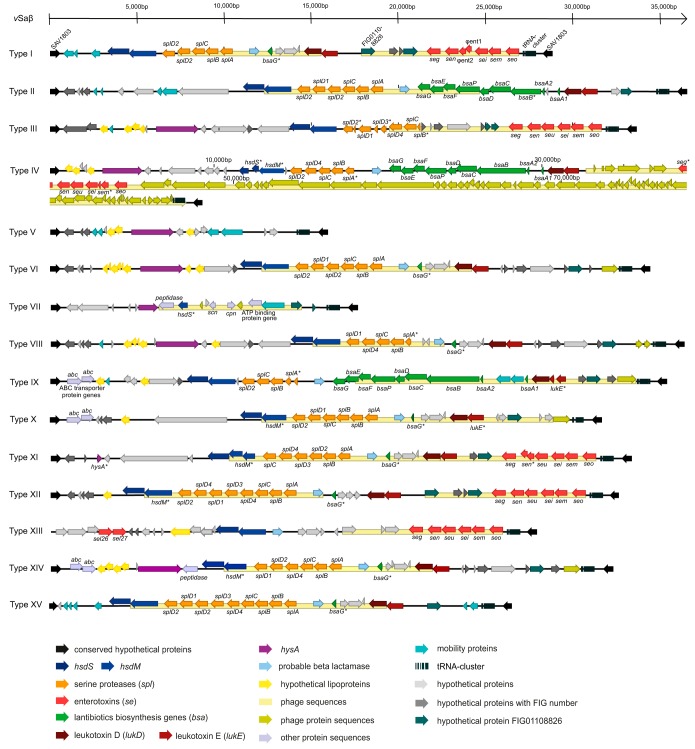
Representation of all Staphylococcus aureus genomic island *v*Saβ types I to XV, their virulence-associated genes, and other hypothetical genes located on *v*Saβ. For each *v*Saβ type, one reference strain is shown. Arrows show orientation of open reading frames. Asterisks indicate truncated or fragmented genes. Note that *v*Saβ IV is substantially longer due to the presence of a complete phage. All other sequences are scaled relative to each other.

All vSaβ islands were enclosed by 2 distinct genes coding for hypothetical proteins (*hp* genes). In the strain Mu50 (reference strain for vSaβ type I [see below]) the *hp* with the locus tag SAV1803 marks the 5′ terminus of vSaβ, and SAV1831 marks the 3′ terminus. These two *hp* genes could be found in nearly all vSaβ islands (see Fig. 1 and Table 1). For convenience, we refer to these sequences as *sav1803* and *sav1831*, respectively. These *hp* genes were highly conserved among all strains, with product amino acid identities of >95% in SAV1803 and >98% in SAV1831. In all strains, *sav1831* directly followed a cluster of 8 tRNAs (tRNA^fMet^, tRNA^Asp^, tRNA^Phe^, tRNA^His^, tRNA^Gly^, tRNA^Asn^, tRNA^Glu^, and tRNA^Ser^).

Most *v*Saβ types followed a similar structural design (with exceptions of types V, VII, and XIII) carrying a distinct set of virulence genes (Fig. 1). They included *hysA*, the lipoprotein (*lpn*) genes, the RM genes *hsdM* and *hsdS*, a cluster of *spl* genes (*splA*, *splB*, *splC*, *splD_1_*, *splD_2_*, *splD_3_*, and *splD_4_*), a probable beta-lactamase gene (*bla*), a cluster of 9 *bsa* genes (*bsaA1*, *bsaA2*, *bsaB*, *bsaC*, *bsaD*, *bsaE*, *bsaF*, *bsaG*, and *bsaP*), the *lukD* and *lukE* genes, as well as an EGC consisting of the *seg*, *sei*, *sem*, *sen*, *seo*, and *seu* genes. In some cases, the *seu* gene was replaced by the two truncated genes, ϕ*ent1* and ϕ*ent2*. The compositions of these genes and gene clusters varied substantially between different *v*Saβ types but were highly conserved within the same *v*Saβ type (Fig. 1 and Table 1). In addition to these core *v*Saβ genes, a number of *hp* genes were found of which some were assigned to a FIG number by the RAST (Rapid Annotations using Subsystem Technology) pipeline.

By typing the *v*Saβ islands and aligning all related sequences, a consensus structure for each *v*Saβ type could be defined. *v*Saβ type I possessed the genes of the type I RM system, an *spl* cluster (*splA*, *splB*, *splC*, and two copies of *splD_2_*), *lukD*, *lukE*, and a complete EGC. The amino acid sequences were, with only very few exceptions, highly similar within this *v*Saβ type (>95%) (Table 1). The exceptions were truncated genes, the lack of a second *splD_2_* gene, and a lower sequence identity of the *splD_2_* protein in three strains (Table 1). Furthermore, 3 sequences for mobile element proteins (*mep*), a *bsaG* fragment, *bla*, and remnants of phages were detected (Table 1; Fig. 1). Comparing the structures of all 11 *v*Saβ type I genomic islands, Mu50 showed consensus and hence was used as a reference for this *v*Saβ type. The consensus of *v*Saβ type II was best represented by S. aureus strain TW20. It harbored the type I RM genes, an *spl* cluster (*splA*, *splB*, *splC*, *splD_1_*, and two copies of *splD_2_*), a *bla*, a *bsa* cluster, and the *lukD* and *lukE* genes. Exceptions were some strains that harbored truncated *hsdM*, *basE*, and *lukE* genes. Moreover, *v*Saβ type II comprised sequences for two *mep* genes, *bla*, a number of *hp* genes, and a partial phage (Table 1). The consensus on *v*Saβ type III was strain MRSA252. It harbored *hysA*, *hsdM*, *hsdS*, an *spl* cluster (*splB*, *splC*, *splD_1_*, *splD_2_*, *splD_3_*, and *splD_4_*), and an EGC. Besides three *spl* genes being all pseudogenes (*splB*, *splD_2_*, and *splD_3_*), *v*Saβ type III lacked *lukD* and *lukE* but had additional genes in its 5′ region (a number of putative *lpn* genes, a *hysA*, and a lipase gene). *v*Saβ type III carried sequences for *mep*, *hp*, and remnants of a phage. In most of the type III strains, the core proteins were highly conserved, showing an identity of ≥99% when S. aureus strain MRSA252 was used as a reference. Deviations thereof could be found in the *spl* cluster and in *hsdM* (Table 1). The *v*Saβ type IV was best represented by strain M3783C and was, with over 85,000 bp, substantially longer than the other *v*Saβ types. It harbored a number of *hp* genes, a *hysA* gene, an *spl* cluster (*splB*, *splC*, *splD_2_*, *splD_4_*, and a truncated *splA*), a complete *bsa* cluster, an EGC (with truncated *seg* and *sem*), *lukD*, *lukE*, and the *bla* gene. *hsdM* and *hsdS* were either missing or had premature stop codons. The amino acid sequences of all other *v*Saβ key genes were highly conserved (≥99%) among strains, with only a small number of exceptions (Table 1). Furthermore, a 50.4-kb intact mosaic phage closest to Ipla88 (NCBI RefSeq accession no. NC011614) was detected in 6 *v*Saβ type IV strains, coding for approximately 75 proteins including those encoded by the EGC also found in other *v*Saβ types. In contrast, the strains M2323C and Sa110 harbored only remnants of two phages; hence, the *v*Saβ islands of these two strains were shorter than the other type IV islands. The structure of *v*Saβ type V differed substantially from the other types and was only 15,965 bp long and highly conserved, showing a nucleotide sequence identity of ≥99% when using strain S0385 as a reference. From the typical genes, *v*Saβ type V harbored only *hysA*. However, it carries a total of 4 transposons, as well as a number of *hp* genes, some of which were related to FIG numbers, or they were identified as *lpn* genes. Within *v*Saβ type VI, strain K2R was a suitable representative. It comprised *hysA*, *hsdS, hsdM*, an *spl* cluster (*splA*, *slpB*, *splC*, *splD_1_*, and two copies of *splD_2_*), *bla*, a *bsaG* fragment, *lukD, lukE*, a phage integrase, as well as a number of *hp* genes, some of which were assigned a FIG number, and others were identified as *lpn* genes. In addition, a partial phage was detected encompassing the entire *spl* cluster. Four strains (M0443, Newbould, G07I, and Lodi4R) had a frameshift in the beginning of *lukE*, leading to a truncated protein. Additionally, G07I and Lodi4R also lacked the *splD_1_* and the second *splD_2_* gene. Except for these very few special cases, all genes of *v*Saβ type VI were highly conserved between strains, showing an amino acid identity of ≥98%. The low overall nucleotide identity of 76% in strain Lodi4R compared to the reference was based on the insertion of the transposon Tn*554* within *hysA*. This strain was, therefore, considered a special case of *v*Saβ type VI. Tn*554* carried 3 transposase genes, as well as a *bla* operon carrying the *blaI*, *blaR1*, and *blaZ*. If Tn*554* was disregarded, the nucleotide sequence identity increased to 93%. *v*Saβ type VII, similar to type V, was short (about 17,460 bp) and lacked the typical *v*Saβ genes, except for *hysA* and a *hsdS* fragment. Furthermore, all *v*Saβ type VII strains showed a partial phage with genes for proteins targeting the host’s immune response (chemotaxis-inhibiting protein Cpn and extracellular complement-binding protein Scn), as well as genes for a peptidase, a phage lysine, an ATP-binding protein, and a transposase. The amino acid sequences of these phage-encoded proteins were identical, with the exception of M3386D that was lacking a part of the phage containing genes coding for the ATP-binding protein and the transposase. The *v*Saβ type VIII included only the JKD6159 strain. It comprised *hysA*, *hsdM, hsdS*, an *spl* cluster (a truncated *splA*, *splB*, *splC*, *splD_1_*, and *splD_4_*), *bla*, a *bsaG* fragment, *lukD, lukE*, a partial phage, a number of *hp* and phage-related genes, and a number of *lpn* genes in the 5′ region. For *v*Saβ type IX, ED133 served as the reference harboring the *v*Saβ key genes *hsdM* and *hsdS*, an *spl* cluster (a truncated *splA*, *slpB*, *splC*, and *splD_2_*), *lukD, lukE* (truncated), *bla*, and a complete *bsa* cluster, as well as a number of *hp* genes, some of which were identified as *lpn* genes and others that were assigned a FIG number. In addition, *v*Saβ type IX carried 2 genes encoding ATP-binding cassette (ABC) transporter proteins. and a gene encoding a phage integrase. Despite the isolates being from 3 different countries, they shared an overall nucleotide sequence identity of ≥95% in their *v*Saβ. The only small difference in the *v*Saβ key genes was found in *hsdM* and *lukD*, which showed lower similarities in some strains (Table 1). *v*Saβ type X showed 93% nucleotide sequence identity. The *v*Saβ consisted of an *spl* cluster (*splA*, two copies of *splB*, *splC*, *splD_1_*, *splD_2_*), a *bla*, a *bsaG* fragment, *lukD*, *lukE*, a complete *hsdS* gene and an *hsdM* gene with a premature stop codon. In addition to a number of *hp* and FIG-assigned genes, *lpn*, two genes encoding an ABC transporter, and a phage integrase gene could be found, similar to *v*Saβ type IX. The amino acid identity in the key genes were ≥98%, with the exceptions of *lukE* being truncated in O11, *splD_1_* lacking in strain O46, and a slightly lower identity in *splD_2_* (93% amino acid identity). *v*Saβ type XI consisted of two strains with an amino acid identity of ≥99% in the vSaβ key genes and a nucleotide identity of 99% over the entire *v*Saβ region. Their *v*Saβ consists of *hsdM* and *hsdS*, but *hsdM* has a premature stop codon. Furthermore, vSaβ type XI was characterized by an *spl* cluster (*splA*, *splB*, *splC*, *splD_2_*, *splD_3_*, and *splD_4_*), a *bla*, a *basG* fragment, *lukD, lukE*, a complete EGC (with a truncated *sen*), several *hp* genes (some of which with assigned FIG numbers), and a short *hysA* fragment. The most striking feature of *v*Saβ type XII was the enlarged *spl* cluster consisting of *splA*, *splB*, *splC*, *splD_1_*, *splD_2_*, *splD_3_*, and 2 copies of *splD_4_*. It further harbored both RM genes (*hsdM* with premature stop codon), *bla*, a *bsaG* fragment, *lukD, lukE*, and a complete EGC along with several *hp* genes, some with assigned FIG numbers. The strains categorized as *v*Saβ type XIII had a typical *v*Saβ structure in the 3′ end, yet a clear beginning could not be defined for this *v*Saβ type, as it lacked the conserved 5′-end *sav1803*. Instead, there were three *hp* genes as well as two recently discovered enterotoxin sequences, *sel26* and *sel27*. These were followed by several *hp* genes (some with assigned FIG number) and genes typical for *v*Saβ, such as *lpn*, *hsdM, hdsS*, *bla*, the EGC followed by the tRNA cluster, and *sav1831,* typically marking the 3′ end. *v*Saβ type XIV harbored *hysA*, both RM genes (*hsdM* and *hsdS*), an *spl* cluster (*splA*, *splB*, *splC*, *splD_1_*, *splD_2_*, and *splD_4_*), *bla*, a *bsaG* fragment, *lukD,* and *lukE*. When strain G08M was used as a reference, most proteins showed an amino acid identity of 100%, with the exceptions being three strains with truncated *hsdM* genes. *v*Saβ type XV consisted of both RM genes (*hsdM* and *hsdS*), an *spl* cluster (*splA*, two copies of *splB*, *splC*, *splD_1_*, two *splD_2_*, *splD_3_*, and *splD_4_*), *bla*, a *bsaG* fragment, *lukD,* and *lukE*. When strain Lodi11bM was used as a reference, the only differences were *lukE* being truncated and SplD_4_ having a lower amino acid identity in strain 4185.

In summary, all *v*Saβ types had the same basic structural design. The design was minimal for types V, VII, and XIII and was more complex for the other types, as they harbored a distinct set of virulence factors and gene clusters. Also common to all *v*Saβ types, except to type XIII, was the pervasive flanking by the same conserved sequences with the locus tags SAV1803 and SAV1831. Variation among the *v*Saβ types was observed for the *spl* cluster in gene number and composition. Furthermore, some types were characterized by truncated RM genes and/or *bsa* or *spl* genes. Within each *v*Saβ type, they were highly conserved, at the structural and the protein levels.

### HsdS.

**FIG 2:**
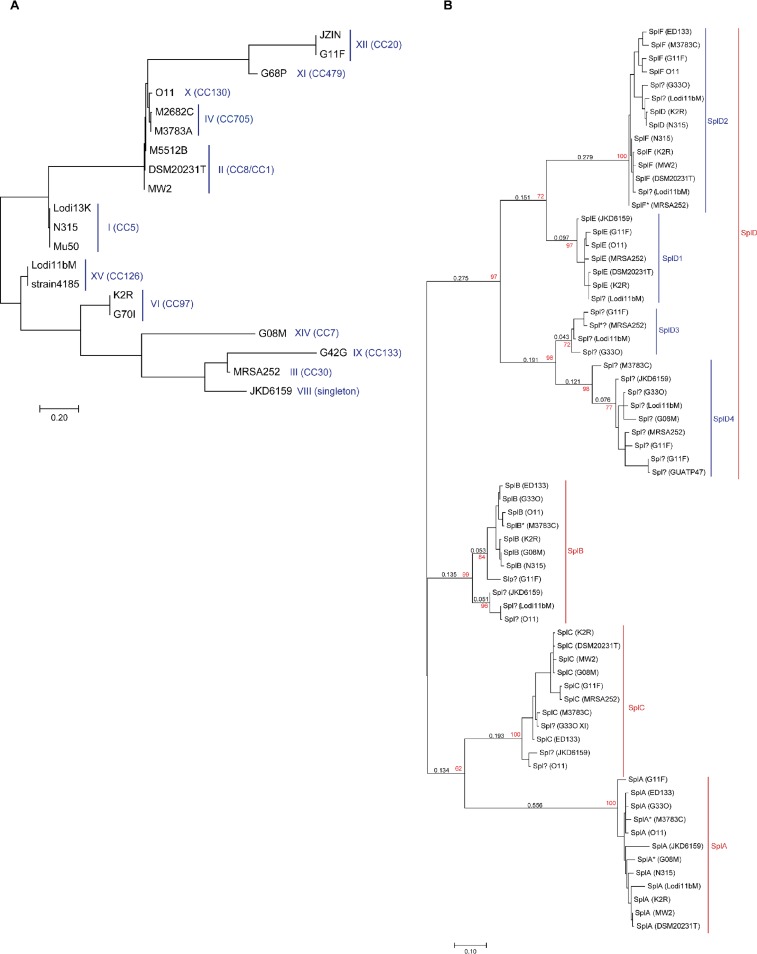
(A and B) Phylogeny of the 20 HsdS (A) and 68 Spl (B) amino acid sequences of Staphylococcus aureus. For both trees, protein sequences that differedin at least one amino acid were aligned, and a phylogenetic tree was reconstructed using maximum likelihood. For each tree, a scale indicates the relative distance on the phylogenetic tree. (A) The blue line indicates a strain’s *v*Saβ type, and its clonal complex (CC) is given in parentheses. (B) The strain from which the amino acid sequence originated is shown in parentheses. Numbers in red represent bootstrap values. Question marks indicate sequences of Spl with previously uncertain or unknown nomenclature, and red and blue lines show suggested nomenclature of the corresponding branch based on phylogenetic distances of the Spl amino acid sequences. Curated sequences are marked with an asterisk.

Inspection of the HsdS sequences with a phylogenetic approach (Fig. 2A) revealed that the HsdS sequence alone does not provide sufficient resolution for inference of gene content or virulence determinants of the entire *v*Saβ. Hence, categorization of *v*Saβ based on the nucleotide sequence identity of the entire genomic island can be considered the method of choice when the entire sequence of the genomic island is available.

### Serine proteases.

All *v*Saβ islands, with the exceptions of types V, VII, and XIII, carried an *spl* cluster, harboring between 4 and 9 *spl* genes. Phylogenetic analyses using the maximum-likelihood method confirmed the existing Spl family members SplA, SplB, SplC, and SplD, but with SplD having 4 different variants (SplD_1_, SplD_2_, SplD_3_, and SplD_4_) (Fig. 2B). SplD_1_ replaces the former SplE, and SplD_2_ replaces the former SplD and SplF. The SplD_3_ and SplD_4_ clades include Spl proteins that could not be assigned into any of the preexisting Spl variants. Furthermore, the presence of multiple *splD* variants per strain points to a relatively recent gene duplication.

### Clonal complexes and *spa* types.

*spa* typing showed that one *v*Saβ type can harbor a number of *spa* types, but each *spa* type is limited to one *v*Saβ type. Highly striking was the consistency of a *v*Saβ type with the strains’ clonality. The predominance of a single clonal complex (CC) per *v*Saβ type underlined the concept that *v*Saβ acquisition and its diversification happened prior or simultaneously to the clonal diversification of ancestral S. aureus strains.

## DISCUSSION

With the rapid advancement of sequencing technologies and the decreasing costs thereof, the amount of S. aureus sequences deposited in databases is growing exponentially. These databases offer a powerful information source for studying the variabilities and consistencies between different isolates and their potential ability to cause disease. As opposed to the slow accumulation of point mutations, acquisition of larger parts of DNA through horizontal gene transfer leads to a rapid genetic change. This can be crucial in survival under certain selection pressures (antibiotics) or in novel niches (new host) (10, 12, 13). The mechanism of how S. aureus acquired its genomic islands is not fully understood, yet here, we have elaborated why phage mediation has played a crucial role.

### Viral origin of *v*Saβ.

Throughout all *v*Saβ types, the strains belonging to one *v*Saβ type almost exclusively harbor the same virulence genes. In addition to the gene composition, an amino acid sequence identity of 95% or higher is another characteristic within each type, and in many cases, even 100% over all strains of a *v*Saβ type (Table 1).

Despite the range of hosts and geographical origins covered in this study, the *v*Saβ islands were always located in between SAV1803 and SAV1831 at the 5′ and 3′ ends, respectively. This highly precise location together with the tRNA cluster preceding SAV1831 as observed throughout all *v*Saβ types strongly indicates that these sites may be key for the presence of the *v*Saβ islands in S. aureus. Indeed, tRNAs are known to harbor a conserved attachment site (14) for integrating prophages and other foreign DNA (15, 16).

It is widely accepted that S. aureus genomic islands were acquired through horizontal gene transfer, but the exact mechanism and their current mobility status have been questioned (8, 10, 17). Our data show the presence of partial phages in almost all *v*Saβ types, with the exceptions of type V, where no phages were predicted, and type IV, where a complete prophage was predicted by PHASTER. In addition, *v*Saβ type IV also harbors all virulence-related key features of *v*Saβ, as follows: the type I RM system, the *spl* cluster, the *bsa* cluster, the leukocidin genes, and the EGC. Other *v*Saβ types possess the EGC only (types I, III, XI, XII, and XIII), the *bsa* cluster only (II and IX), or lack both. In this sense, *v*Saβ type IV can be considered the most complete of all *v*Saβ types regarding virulence-related gene content and phage. Moon et al. (14) showed that this very phage of strain RF122 (*v*Saβ type IV) can be mobilized *in vitro*, resulting in heterogeneous, yet overlapping particles that integrated sequentially through recombination into the host; in some cases, it even resulted in the transfer of the almost-complete *v*Saβ (14).

Our results also demonstrate that all *v*Saβ islands containing the key virulence factors also harbored partial phages, suggesting that *v*Saβ originated in an ancestral S. aureus strain through a phage integration event. In the course of evolution, multiple recombination, integration, and excision events may have occurred, resulting in various combinations of the virulence genes that we now observe in the different *v*Saβ types. To our knowledge, *v*Saβ type IV is the only type to harbor a complete phage that has been shown to be mobilized, suggesting that the phages of the other *v*Saβ types are likely to have lost their mobility in the course of evolution.

The unequivocal location, the demonstrated mobility (14), and the ubiquitous presence of phage particles in *v*Saβ islands, as well as a conserved phage attachment site, strongly support the hypothesis that *v*Saβ was mediated by a phage, followed by diversification into the types observed today.

### *v*Saβ and clonality.

In total, *v*Saβ typing of 103 S. aureus strains revealed 15 different types. They correlated strongly with the CCs and were always linked to one specific *v*Saβ type. Therefore, the CC of S. aureus allows the link to virulence-associated *v*Saβ key genes. This link has been made previously (18), but by assessing and categorizing the novel 12 *v*Saβ types, we confirmed this observation to be a general principle. Indeed, all studied strains follow this pattern, indicating that the horizontal acquisition of *v*Saβ in an ancestral S. aureus strain happened before divergence into the clones occurred. It is evident that the primordial *v*Saβ underwent multiple genetic changes (e.g., recombination and duplications), likely just prior or simultaneously to clone formation, resulting in the types observed today.

The evolutionary link of *v*Saβ types and CCs are in perfect agreement with the topology of the phylogenetic tree by Boss et al. (19). For that study, the authors concatenated 7 S. aureus-specific genes from 30 different strains and aligned these using the Needleman-Wunsch algorithm. This alignment was then used to construct a maximum parsimony phylogeny. This phylogeny showed the evolutionary relationship between some of the most common S. aureus CCs that all possess a *v*Saβ and a type-specific set of virulence genes. Therefore, it is evident that the common ancestor of these CCs already carried an ancestral *v*Saβ.

The discrepancy between the overall structures of *v*Saβ types V and VII and all other *v*Saβ types can be explained by an evolutionary loss of the *v*Saβ key genes in these types during two separate events. We can still find *v*Saβ typical elements, such as the conserved regions marking the 5′ and 3′ ends of *v*Saβ or the almost ubiquitous *hp* FIG01108826 in the 3′ region. Furthermore, type V harbors sequences that can be found in a number of other *v*Saβ types, such as 3 *hp* genes with an assigned FIG number located the 5′ region (FIG01108398, FIG01108644, and FIG01108514), *lpn*, and *hysA*. Interestingly, type VII encodes different virulence factors that tackle the host’s immune defenses. As *v*Saβ types V and VII have additional transposase genes, we suggest that in these two types, the *v*Saβ key genes were replaced by a transposon, indicating that *v*Saβ themselves may be a hot spot for inserting mobile genetic elements and potentially accumulating virulence factors.

*v*Saβ type XIII has a deviate structure, as it lacks the typical 5′ region found in the other types. Together with types V and VII, it lacks the *spl* cluster and the *luk* genes. We cannot exactly locate the beginning of this *v*Saβ type on the genome, but interestingly, we found two very recently discovered phage-associated enterotoxin genes (*sel26* and *sel27*) in that region (20). Furthermore, we found an *hp* (FIG01108398) which is adjacent to the SAV1803 in some other types.

### Virulence and *v*Saβ.

The variable regions contribute to the fate of an S. aureus strain on a given host and its disease-causing potential, as these regions encode a number of virulence factors (6, 10). They include the pore-forming LukD and LukE, which are present on most *v*Saβ types (except III, V, VII, and XIII). Both proteins are members of the leukocidin family (21) that form pores in the lipid bilayer of host cells, particularly in neutrophils, leading to cell death and, therefore, the promotion of immune evasion and progression of the infection (22, 23).

Further virulence factors encoded on *v*Saβ are the Spl proteins that are unique to S. aureus and are organized in an operon (11). *v*Saβ types V, VII, and XIII lack the *spl* cluster, whereas all other types have a *spl* cluster, some of them with truncated genes. Based on our phylogenetic approach, there are 4 different *spl* genes (*splA*, *splB*, *splC*, and *splD*) and 4 gene variants of *splD* (*splD_1_*, *splD_2_*, *splD_3_*, and *splD_4_*). SplA, SplB, and SplC form their own clades and are clearly separated from the SplD clade (Fig. 2B). The former SplD and SplF are now members of the SplD_2_ clade, which is unsurprising, as their amino acid identity can be as high as 94% (11, 24). The SplD_3_ and SplD_4_ clades consist both of Spl sequences that have not been previously studied and did not match any of the predefined *spl* genes based on sequence similarity. We observed a high conservation of the *spl* operon within but not between *v*Saβ types. The function of *spl* genes in infection and disease is still largely unclear (24–27). Studies showed that *spl* genes are expressed and secreted during host infection (25, 27) and have been linked to allergic reactions (28, 29). Interestingly, *v*Saβ can harbor multiple copies of the very same *spl* gene. The plasticity of the *spl* cluster is an evidence for its varied importance among different *v*Saβ types.

Hyaluronic acid is a major component of the connective tissue, in particular, the extracellular matrix (30). HysA is considered a virulence factor, as it can depolymerize hyaluronic acid and favoring the spreading of infection (31, 32). Many S. aureus genomes contain a chromosomal copy of *hysA* (33) outside of *v*SAβ. The additional copy present in the *v*Saβ region of types III to VIII and XIV may be linked to enhanced invasiveness of these clones.

Lantibiotics (or bacteriocins) are antimicrobial peptides produced by some Gram-positive bacteria against closely related species (34, 35) and are thought to play a role in colonization by outcompeting other bacteria (10, 24).

### *v*Saβ typing.

In contrast to the link of CC and *v*Saβ types, it has been suggested that the *v*Saβ type is dependent on the strain’s HsdS sequence (8). These predictions were not very reliable, as they were based on 3 *v*Saβ types and 12 sequenced S. aureus strains only. From a total of 15 *v*Saβ types, we defined 12 new types based on multiple hosts and geographical regions. Within the scope of our study, the method used for typing the *v*Saβ region proved to be a very robust procedure, as all used strains could be exclusively allocated to one *v*Saβ type, and cross-classification was never observed. Hence, we expect the specified principles to be robust enough to uphold future findings when additional S. aureus strains are analyzed.

In the scope of this study, we limited the data set to clinically relevant S. aureus strains either from human or animal infections. Strains that were not invasive (i.e., colonizers and strains isolated from foods) were excluded from the data set. Hence, our results are limited to these invasive strains only, yet we did find that a few colonizers also fit into the system proposed here (data not shown). In the future, more *v*Saβ regions need to be analyzed spanning more hosts and including noninvasive strains.

### Conclusions.

In general, the *v*Saβ islands harbor a number of virulence-associated and pathogenic genes with different scopes of action. While the exact functions of many of these genes are yet to be unraveled, it is clear that they have severe effects on the host’s health and are likely to play key roles in S. aureus adaptation to the clinical microenvironment. Our data support a viral origin of the *v*Saβ region. As the *v*Saβ type is strongly linked to a strain’s CC, acquisition of the *v*Saβ region happened in a very ancestral S. aureus strain while the transformation to the distinct vSaβ subtypes occurred before or simultaneously to diversification into the different clones. The here-suggested superordinate system to classify S. aureus strains based on their *v*Saβ region may be used in the future to assess the clinical potential of an S. aureus strain. In the future, more *v*Saβ genomic islands need to be analyzed and categorized into this superordinate system.

## MATERIALS AND METHODS

### Sequencing.

The in-house S. aureus genome collection includes 23 strains that were previously sampled from bovine mastitis (36). All strains were kept in skim milk at –20°C and were recultured at 37°C for 24 h on blood agar (bioMérieux Suisse s.a., Geneva, Switzerland). Plates were sent to Microsynth AG (Balgach, Switzerland) for DNA extraction and subsequent whole-genome sequencing (WGS), initially by the 454 (Roche, Basel, Switzerland) and later by the Illumina (Illumina, Inc., San Diego, CA) technology, as the 454 method was no longer available. For *de novo* assembly of the reads to contigs, they used the Newbler v.2.6 assembler for the 454 (Roche) technology and SPAdes v.3.1 (37) for the Illumina technology (see File S1 in the supplemental material for details). In addition, contigs of 4 bovine S. aureus strains after Illumina WGS were provided by M. Luini (IZSLER, Lodi, Italy) and P. Cremonesi (CNR, Lodi, Italy).

### Data collection.

In an attempt to characterize the *v*Saβ islands of our 27 bovine mastitis S. aureus genomes as described by Baba et al. (8), it turned out that many of them did not match the 3 previously defined types proposing a much higher diversity of *v*Saβ; hence, an in-depth characterization based on more data was required. To do so, more sequences were collected by a BLAST search (36) of each known, presumptively novel, *v*Saβ against the NCBI nonredundant/nucleotide (nr/nt) database (https://www.ncbi.nlm.nih.gov/nucleotide/) using the default BLAST settings and limiting the search to S. aureus. To increase the diversity of the *v*Saβ islands, the NCBI databases were also evaluated for genomes or contigs of clinical S. aureus strains from hosts other than humans or cattle. This approach was selected, as invasive strains of S. aureus are host specific (19, 38, 39), possibly accounting for additional types of *v*Saβ islands. For each *v*Saβ type, ≥10 chromosomal sequences of clinical S. aureus were then attempted to be retrieved. If this was impossible, the BLAST search was further extended to the NCBI whole genome shotgun contig database (https://www.ncbi.nlm.nih.gov/assembly/). All available chromosomal sequences were then retrieved, including all contigs containing a complete *v*Saβ region.

Using this approach, a total of 76 sequences were obtained, with 43 sequences from genomes and 33 sequences from contigs (see File S2).

### Data analysis.

**(i) Data set.** In total, 27 of our own and 76 publicly available sequences were included in the present study. From these 103 analyzed S. aureus strains, 58 originated from human hosts, 33 were from bovine hosts, and 7 were from ovine hosts. For 5 strains, no host information was available. Of these 103 strains, 37 strains showed known *v*Saβ types (I to III), and 66 strains showed novel *v*Saβ types (IV to XV) (see File S2 for details).

**(ii) *v*Saβ typing.** The *v*Saβ regions were identified on genome sequences by aligning the sequences of conserved *hp* genes flanking *v*Saβ at the 5′ end (SAV1803) and 3′ end (SAV1831) (8) using the Clone Manager Professional 9 (CM9) software (Scientific & Educational Software, Denver, CO). Subtyping of the *v*Saβ islands was then based on the initial work by Baba et al. (8). Using the *hsdS* gene of 12 clinically relevant S. aureus strains, Baba et al. grouped them into three *v*Saβ types (*v*Saβ I, *v*Saβ II, and *v*Saβ III [8]). We then aligned the *v*Saβ sequences of these 12 strains in the CM9 software using the Needleman-Wunsch algorithm and found that the overall similarities within a *v*Saβ type were ≥90%. For each of these *v*Saβ types, a representative sequence was then selected and considered the type-specific reference sequence (Table 1). Disregarding included phages and transposons, *v*Saβ islands showing overall sequence similarities of <90% compared to all the existing reference sequences were then considered new *v*Saβ types. Through the iterative process of aligning nontyped *v*Saβ islands to each of the *v*Saβ type reference sequences, the 103 sequences of the data set were grouped into 15 *v*Saβ types (*v*Saβ I to XV, Table 1).

**(iii) HsdS phylogenetics.** To infer the phylogeny of the HsdS proteins (37, 40), the corresponding nucleotide sequences were translated using the standard code. Proteins that differed in at least one amino acid were then used for a multiple-sequence alignment (MSA) in the CM9 software using the Needleman-Wunsch approach. The MSA was exported to the BioEdit software (http://www.mbio.ncsu.edu/BioEdit/bioedit.html) for visual inspection and manual curation. Afterwards, the curated MSA was imported into the MEGA X software (41) to assess a maximum likelihood (ML) phylogeny of the HsdS proteins. To do so, models using 9 different substitution matrices were computed including or leaving out modeling for invariant sites and for the evolutionary rate differences among sites by a discrete gamma distribution. The optimal model was then selected based on the lowest value of the Akaike information criterion resulting in the JTT substitution matrix (37), with specific parameters for the gamma distribution. This model was then used to construct a phylogenetic ML tree (MEGA X software [41]). Initial trees were built by applying the maximum parsimony algorithm. The topology with the highest log-likelihood value was then selected (Fig. 2A). Strains lacking the *hsdS* gene were excluded.

**(iv) Multilocus and *spa* typing.** Multilocus sequence typing (MLST) of seven housekeeping genes (42) and *spa* typing (43) were performed for all S. aureus genomes using the Center for Genomic Epidemiology online platform (http://www.genomicepidemiology.org/). In the eBURST V3 program, the sequence types (STs) resulting from the MLST were used to allocate each strain to a clonal complex (CC).

**(v) Annotations.** Annotations were assigned to the *v*Saβ regions using the RAST pipeline (44–46). RAST annotations of all genes (including *hp* genes with or without a FIG number assigned by the RAST pipeline) were verified by a BLAST search against the UniProt database (47). Where necessary, annotations were corrected manually based on the UniProt data; whenever possible, reviewed entries were used. If the RAST pipeline failed to annotate genes known to be located on *v*Saβ, these genes were searched manually by aligning the nucleotide sequences of the well-characterized strains N315 and MW2 to the *v*Saβ of interest. Transfer RNAs were identified by tRNAscan-SE 2.0 (48).

**(vi) Phage detection.** Phages and phage remnants were identified by the online software Phage Search Tool Enhanced Release (PHASTER) (49, 50). To ensure detection, regions 5,000 bp up- and downstream of *v*Saβ were included in the analysis.

**(vii) Serine protease nomenclature.** All complete and curated *spl* sequences were analyzed and translated into proteins using the standard code. Sixty-eight amino acid sequences were unique (i.e., differed in at least one amino acid) and were used to reconstruct the phylogeny using the same methods as mentioned above for the HsdS protein sequence (ML method, JTT substitution matrix, discrete gamma distribution to model evolutionary rate differences among sites) (Fig. 2A and B). As the current nomenclature of *spl* does not account for the phylogenetic relationship between their sequences and in some cases is ambiguous and arbitrary, a new nomenclature was developed using the ML phylogenetic approach based on the protein sequences. Starting from the root of the phylogenetic tree, each node along a branch is followed until a node forming a monophyletic clade is reached whose branch connecting to its predecessor is characterized by a bootstrap value (BT) of ≥95 and a phylogenetic distance (*d*) of >0.10. All leaves forming this clade obtain the same letter (e.g., “D”) added to the basic name of the protein (in this case “Spl”), resulting in the name SplD for all of these leaves. The branch is then followed toward the leaves. If a node is reached forming a monophyletic subclade whose branch connecting to the predecessor is characterized by a BT of ≥95 and *d* of >0.10, the leaves of this subclade are labeled by adding a number (e.g., “2”), resulting in the name SplD2. At this stage, further evaluation toward the leaves is stopped. If only one branch of a bifurcation fulfills these conditions, the subclade formed by the nonfulfilling branch gets a new letter or number as well.

### Data availability.

All whole-genome projects have been deposited in the NCBI database under the BioProject number PRJNA531079 with the accession numbers SZYI00000000 (Lodi13K), SZYM00000000 (G12B), SZYO00000000 (G29N), SZYT00000000 (Lodi10B), VCQP00000000 (M2084B), SZYY00000000 (M2130B), SZZA00000000 (M2529B), SZZF00000000 (M5512B), VCQQ00000000 (M5171B), SZZG00000000 (M6020B), SZZE00000000 (M3783C), SZZC00000000 (M2839C), SZYW00000000 (M1280C), SZYX00000000 (M1655C), SZYZ00000000 (M2323C), SZZB00000000 (M2682A), SZYS00000000 (K2R), SZYJ00000000 (G07I), SZYV00000000 (Lodi4R), SZZD00000000 (M3386D), SZYQ00000000 (G42G), SZYP00000000 (G33O), SZYR00000000 (G68P), SZYL00000000 (G11F), SZYN00000000 (G19F), SZYK00000000 (G08M), and SZYU00000000 (Lodi11bM).

## Supplementary Material

Supplemental file 1

Supplemental file 2

## References

[1] Peacock SJ, De Silva I, Lowy FD 2001 What determines nasal carriage of *Staphylococcus aureus*? Trends Microbiol 9:605–610. doi:10.1016/S0966-842X(01)02254-5. 11728874

[2] Kluytmans J, Belkum A, Verbrugh H 1997 Nasal carriage of *Staphylococcus aureus*: epidemiology, underlying mechanisms, and associated risks. Clin Microbiol Rev 10:505–520. doi:10.1128/CMR.10.3.505. 9227864 PMC172932

[3] Herrmann M, Smeltzer MS 2016 Clinical significance in humans, p 23–43. *In* Somerville GA (ed), Staphylococcus genetics and physiology, 1st ed Caister Academic Press, Norfolk, United Kingdom.

[4] Loy JD 2016 Staphylococcus: clinical significance in animals, p 45–66. *In* Somerville GA (ed), Staphylococcus genetics and physiology, 1st ed Caister Academic Press, Norfolk, United Kingdom.

[5] Lowy FD 1998 *Staphylococcus aureus* Infections. N Engl J Med 339:520–532. doi:10.1056/NEJM199808203390806. 9709046

[6] Malachowa N, Deleo FR 2010 Mobile genetic elements of *Staphylococcus aureus*. Cell Mol Life Sci 67:3057–3071. doi:10.1007/s00018-010-0389-4. 20668911 PMC2929429

[7] Lindsay JA, Holden M 2004 *Staphylococcus aureus*: superbug, super genome? Trends Microbiol 12:378–385. doi:10.1016/j.tim.2004.06.004. 15276614

[8] Baba T, Bae T, Schneewind O, Takeuchi F, Hiramatsu K 2008 Genome sequence of *Staphylococcus aureus* strain Newman and comparative analysis of staphylococcal genomes: polymorphism and evolution of two major pathogenicity islands. J Bacteriol 190:300–310. doi:10.1128/JB.01000-07. 17951380 PMC2223734

[9] Fitzgerald JR, Monday SR, Foster TJ, Bohach GA, Hartigan PJ, Meaney WJ, Smyth CJ 2001 Characterization of a putative pathogenicity island from bovine *Staphylococcus aureus* encoding multiple superantigens. J Bacteriol 183:63–70. doi:10.1128/JB.183.1.63-70.2001. 11114901 PMC94850

[10] Baba T, Takeuchi F, Kuroda M, Yuzawa H, Aoki KI, Oguchi A, Nagai Y, Iwama N, Asano K, Naimi T, Kuroda H, Cui L, Yamamoto K, Hiramatsu K 2002 Genome and virulence determinants of high virulence community-acquired MRSA. Lancet 359:1819–1827. doi:10.1016/S0140-6736(02)08713-5. 12044378

[11] Reed SB, Wesson CA, Liou LE, Trumble WR, Schlievert PM, Bohach GA, Bayles KW 2001 Molecular characterization of a novel *Staphylococcus aureus* serine protease operon. Infect Immun 69:1521–1527. doi:10.1128/IAI.69.3.1521-1527.2001. 11179322 PMC98051

[12] Rao RT, Shofia SI, Manna A, Jayakumar K 2016 An account of genomic islands of zoonotic origin Staphylococcus aureus genomes–*in silico* approach. 2016 International Conference on Bioinformatics and Systems Biology, 4 to 6 March 2016, Allahabad, India.

[13] Méric G, Miragaia M, De Been M, Yahara K, Pascoe B, Mageiros L, Mikhail J, Harris LG, Wilkinson TS, Rolo J, Lamble S, Bray JE, Jolley KA, Hanage WP, Bowden R, Maiden MCJ, Mack D, De Lencastre H, Feil EJ, Corander J, Sheppard SK 2015 Ecological overlap and horizontal gene transfer in *Staphylococcus aureus* and *Staphylococcus epidermidis*. Genome Biol Evol 7:1313–1328. doi:10.1093/gbe/evv066. 25888688 PMC4453061

[14] Moon BY, Park JY, Hwang SY, Robinson DA, Thomas JC, Fitzgerald JR, Park YH, Seo KS 2015 Phage-mediated horizontal transfer of a *Staphylococcus aureus* virulence-associated genomic island. Sci Rep 5:9784. doi:10.1038/srep09784. 25891795 PMC4402969

[15] Hacker J, Kaper JB 2000 Pathogenicity islands and the evolution of microbes. Annu Rev Microbiol 54:641–679. doi:10.1146/annurev.micro.54.1.641. 11018140

[16] Darmon E, Leach D 2014 Bacterial genome instability. Microbiol Mol Biol Rev 78:1–39. doi:10.1128/MMBR.00035-13. 24600039 PMC3957733

[17] Dobrindt U, Hochhut B, Hentschel U, Hacker J 2004 Genomic islands in pathogenic and environmental microorganisms. Nat Rev Microbiol 2:414–424. doi:10.1038/nrmicro884. 15100694

[18] Lindsay JA, Moore CE, Day NP, Peacock SJ, Witney AA, Stabler RA, Husain SE, Butcher PD, Hinds J 2006 Microarrays reveal that each of the ten dominant lineages of *Staphylococcus aureus* has a unique combination of surface-associated and regulatory genes. J Bacteriol 188:669–676. doi:10.1128/JB.188.2.669-676.2006. 16385056 PMC1347281

[19] Boss R, Cosandey A, Luini M, Artursson K, Bardiau M, Breitenwieser F, Hehenberger E, Lam T, Mansfeld M, Michel A, Mösslacher G, Naskova J, Nelson S, Podpečan O, Raemy A, Ryan E, Salat O, Zangerl P, Steiner A, Graber HU 2016 Bovine *Staphylococcus aureus*: subtyping, evolution, and zoonotic transfer. J Dairy Sci 99:515–528. doi:10.3168/jds.2015-9589. 26601578

[20] Zhang DF, Yang XY, Zhang J, Qin X, Huang X, Cui Y, Zhou M, Shi C, French NP, Shi X 2018 Identification and characterization of two novel superantigens among *Staphylococcus aureus* complex. Int J Med Microbiol 308:438–446. doi:10.1016/j.ijmm.2018.03.002. 29574061

[21] Gravet A, Colin DA, Keller D, Girardot R, Monteil H, Prévost G, Giradot R 1998 Characterization of a novel structural member, LukE-LukD, of the bi-component staphylococcal leucotoxins family. FEBS Lett 436:202–208. doi:10.1016/s0014-5793(98)01130-2. 9781679

[22] Alonzo F, Torres VJ 2014 The bicomponent pore-forming leucocidins of *Staphylococcus aureus*. Microbiol Mol Biol Rev 78:199–230. doi:10.1128/MMBR.00055-13. 24847020 PMC4054254

[23] Alonzo F III, Benson MA, Chen J, Novick RP, Shopsin B, Torres TJ 2012 *Staphylococcus aureus* leukocidin ED contributes to systemic infection by targeting neutrophils and promoting bacterial growth in vivo. Mol Microbiol 83:423–435. doi:10.1111/j.1365-2958.2011.07942.x. 22142035 PMC3258504

[24] Stach N, Kaszycki P, Wladyka B, Dubin G 2018 Extracellular proteases of Staphylococcus spp, p 135–145. *In* Savini V (ed), Pet-to-man travelling staphylococci. Elsevier, Inc, Dublin, Ireland.

[25] Paharik AE, Salgado-Pabon W, Meyerholz DK, White MJ, Schlievert PM, Horswill AR 2016 The Spl serine proteases modulate *Staphylococcus aureus* protein production and virulence in a rabbit model of pneumonia. mSphere 1:e00208-16. doi:10.1128/mSphere.00208-16. 27747296 PMC5061998

[26] Kolar SL, Antonio Ibarra J, Rivera FE, Mootz JM, Davenport JE, Stevens SM, Horswill AR, Shaw LN 2013 Extracellular proteases are key mediators of *Staphylococcus aureus* virulence via the global modulation of virulence-determinant stability. Microbiologyopen 2:18–34. doi:10.1002/mbo3.55. 23233325 PMC3584211

[27] Zdzalik M, Karim AY, Wolski K, Buda P, Wojcik K, Brueggemann S, Wojciechowski P, Eick S, Calander AM, Jonsson IM, Kubica M, Polakowska K, Miedzobrodzki J, Wladyka B, Potempa J, Dubin G 2012 Prevalence of genes encoding extracellular proteases in *Staphylococcus aureus*–important targets triggering immune response in vivo. FEMS Immunol Med Microbiol 66:220–229. doi:10.1111/j.1574-695X.2012.01005.x. 22762789

[28] Stentzel S, Teufelberger A, Nordengrün M, Kolata J, Schmidt F, van Crombruggen K, Michalik S, Kumpfmüller J, Tischer S, Schweder T, Hecker M, Engelmann S, Völker U, Krysko O, Bachert C, Bröker BM 2017 Staphylococcal serine protease-like proteins are pacemakers of allergic airway reactions to *Staphylococcus aureus*. J Allergy Clin Immunol 139:492–500. doi:10.1016/j.jaci.2016.03.045. 27315768

[29] Teufelberger AR, Nordengrün M, Braun H, Maes T, De Grove K, Holtappels G, O'Brien C, Provoost S, Hammad H, Gonçalves A, Beyaert R, Declercq W, Vandenabeele P, Krysko DV, Bröker BM, Bachert C, Krysko O 2018 The IL-33/ST2 axis is crucial in type 2 airway responses induced by *Staphylococcus aureus*-derived serine protease-like protein D. J Allergy Clin Immunol 141:549–559. doi:10.1016/j.jaci.2017.05.004. 28532656

[30] Necas J, Bartosikova L, Brauner P, Kolar J 2008 Hyaluronic acid (hyaluronan): a review. Vet Med (Praha) 53:397–411. doi:10.17221/1930-VETMED.

[31] Makris G, Wright JD, Ingham E, Holland KT 2004 The hyaluronate lyase of *Staphylococcus aureus*–a virulence factor? Microbiology 150:2005–2013. doi:10.1099/mic.0.26942-0. 15184586

[32] Ibberson CB, Jones CL, Singh S, Wise MC, Hart ME, Zurawski DV, Horswill AR 2014 *Staphylococcus aureus* hyaluronidase is a CodY-regulated virulence factor. Infect Immun 82:4253–4264. doi:10.1128/IAI.01710-14. 25069977 PMC4187871

[33] Farrell AM, Taylor D, Holland KT 1995 Cloning, nucleotide-sequence determination and expression of the *Staphylococcus aureus* hyaluronate lyase gene. FEMS Microbiol Lett 130:81–85. doi:10.1111/j.1574-6968.1995.tb07702.x. 7557301

[34] McAuliffe O, Ross RP, Hill C 2001 Lantibiotics: biosynthesis and mode of action. FEMS Microbiol Lett 25:285–308. doi:10.1111/j.1574-6976.2001.tb00579.x. 11348686

[35] Daly KM, Upton M, Sandiford SK, Draper LA, Wescombe PA, Jack RW, O'Connor PM, Rossney A, Götz F, Hill C, Cotter PD, Ross RP, Tagg JR 2010 Production of the Bsa lantibiotic by community-acquired *Staphylococcus aureus* strains. J Bacteriol 192:1131–1142. doi:10.1128/JB.01375-09. 20023032 PMC2812960

[36] Fournier C, Kuhnert P, Frey J, Miserez R, Kirchhofer M, Kaufmann T, Steiner A, Graber HU 2008 Bovine *Staphylococcus aureus*: association of virulence genes, genotypes and clinical outcome. Res Vet Sci 85:439–448. doi:10.1016/j.rvsc.2008.01.010. 18358507

[37] Roberts GA, Houston PJ, White JH, Chen K, Stephanou AS, Cooper LP, Dryden DTF, Lindsay JA 2013 Impact of target site distribution for type I restriction enzymes on the evolution of methicillin-resistant *Staphylococcus aureus* (MRSA) populations. Nucleic Acids Res 41:7472–7484. doi:10.1093/nar/gkt535. 23771140 PMC3753647

[38] Grundmann H, Aanensen DM, Van Den Wijngaard CC, Spratt BG, Harmsen D, Friedrich AW, Sabat AJ, Muilwijk J, Monen J, Tami A, Donker T, Mittermayer H, Krziwanek K, Stumvoll S, Koller W, Denis O, Struelens M, Nashev D, Budimir A, Kalenic S, Pieridou-Bagatzouni D, Jakubu V, Zemlickova H, Westh H, Sørum M, Skov R, Laurent F, Ettienne J, Strommenger B, Witte W, Vourli S, Vatopoulos A, Vainio A, Vuopio-Varkila J, Fuzi M, Ungvári E, Murchan S, Rossney A, Miklasevics E, Balode A, Haraldsson G, Kristinsson KG, Monaco M, Pantosti A, Borg M, Van Santen-Verheuvel M, Huijsdens X, Marstein L, Jacobsen T, Simonsen GS, 2010 Geographic distribution of *Staphylococcus aureus* causing invasive infections in Europe: a molecular-epidemiological analysis. PLoS Med 7:e1000215. doi:10.1371/journal.pmed.1000215. 20084094 PMC2796391

[39] Leuenberger A, Sartori C, Boss R, Resch G, Oechslin F, Steiner A, Moreillon P, Graber HU 2019 Genotypes of *Staphylococcus aureus*: on-farm epidemiology and the consequences for prevention of intramammary infections. J Dairy Sci 102:3295–3309. doi:10.3168/jds.2018-15181. 30738682

[40] Lindsay JA 2010 Genomic variation and evolution of *Staphylococcus aureus*. Int J Med Microbiol 300:98–103. doi:10.1016/j.ijmm.2009.08.013. 19811948

[41] Kumar S, Stecher G, Li M, Knyaz C, Tamura K 2018 MEGA X: Molecular Evolutionary Genetics Analysis across computing platforms. Mol Biol Evol 35:1547–1549. doi:10.1093/molbev/msy096. 29722887 PMC5967553

[42] Larsen MV, Cosentino S, Rasmussen S, Friis C, Hasman H, Marvig RL, Jelsbak L, Sicheritz-Pontén T, Ussery DW, Aarestrup FM, Lund O 2012 Multilocus sequence typing of total-genome-sequenced bacteria. J Clin Microbiol 50:1355–1361. doi:10.1128/JCM.06094-11. 22238442 PMC3318499

[43] Bartels MD, Petersen A, Worning P, Nielsen JB, Larner-Svensson H, Johansen HK, Andersen LP, Jarløv JO, Boye K, Larsen AR, Westh H 2014 Comparing whole-genome sequencing with Sanger sequencing for spa typing of methicillin-resistant *Staphylococcus aureus*. J Clin Microbiol 52:4305–4308. doi:10.1128/JCM.01979-14. 25297335 PMC4313303

[44] Aziz RK, Bartels D, Best A, DeJongh M, Disz T, Edwards RA, Formsma K, Gerdes S, Glass EM, Kubal M, Meyer F, Olsen GJ, Olson R, Osterman AL, Overbeek RA, McNeil LK, Paarmann D, Paczian T, Parrello B, Pusch GD, Reich C, Stevens R, Vassieva O, Vonstein V, Wilke A, Zagnitko O 2008 The RAST server: Rapid Annotations using Subsystems Technology. BMC Genomics 9:75. doi:10.1186/1471-2164-9-75. 18261238 PMC2265698

[45] Overbeek R, Olson R, Pusch GD, Olsen GJ, Davis JJ, Disz T, Edwards RA, Gerdes S, Parrello B, Shukla M, Vonstein V, Wattam AR, Xia F, Stevens R 2014 The SEED and the Rapid Annotation of microbial genomes using Subsystems Technology (RAST). Nucleic Acids Res 42:206–214. doi:10.1093/nar/gkt1226. PMC3965101 24293654

[46] Brettin T, Davis JJ, Disz T, Edwards RA, Gerdes S, Olsen GJ, Olson R, Overbeek R, Parrello B, Pusch GD, Shukla M, Thomason JA, Stevens R, Vonstein V, Wattam AR, Xia F 2015 RASTtk: a modular and extensible implementation of the RAST algorithm for building custom annotation pipelines and annotating batches of genomes. Sci Rep 5:8365. doi:10.1038/srep08365. 25666585 PMC4322359

[47] Bateman A, Martin MJ, O’Donovan C, Magrane M, Alpi E, Antunes R, Bely B, Bingley M, Bonilla C, Britto R, Bursteinas B, Bye-AJee H, Cowley A, Da Silva A, De Giorgi M, Dogan T, Fazzini F, Castro LG, Figueira L, Garmiri P, Georghiou G, Gonzalez D, Hatton-Ellis E, Li W, Liu W, Lopez R, Luo J, Lussi Y, MacDougall A, Nightingale A, Palka B, Pichler K, Poggioli D, Pundir S, Pureza L, Qi G, Rosanoff S, Saidi R, Sawford T, Shypitsyna A, Speretta E, Turner E, Tyagi N, Volynkin V, Wardell T, Warner K, Watkins X, Zaru R, Zellner H, Xenarios I, 2017 UniProt: the universal protein knowledgebase. Nucleic Acids Res 45:D158–D169. doi:10.1093/nar/gkw1099. 27899622 PMC5210571

[48] Lowe TM, Chan P 2016 tRNAscan-SE On-line: integrating search and context for analysis of transfer RNA genes. Nucleic Acids Res 44:W54–W57. doi:10.1093/nar/gkw413. 27174935 PMC4987944

[49] Zhou Y, Liang Y, Lynch KH, Dennis JJ, Wishart DS 2011 PHAST: a fast phage search tool. Nucleic Acids Res 39:W347–W352. doi:10.1093/nar/gkr485. 21672955 PMC3125810

[50] Arndt D, Grant JR, Marcu A, Sajed T, Pon A, Liang Y, Wishart DS 2016 PHASTER: a better, faster version of the PHAST phage search tool. Nucleic Acids Res 44:W16–W21. doi:10.1093/nar/gkw387. 27141966 PMC4987931

